# Combining body mass index with waist circumference to assess coronary microvascular function in patients with non-obstructive coronary artery disease

**DOI:** 10.1007/s12350-021-02788-3

**Published:** 2021-09-03

**Authors:** Ruonan Wang, Xiang Li, Shihao Huangfu, Qi Yao, Ping Wu, Zhifang Wu, Li Li, Yuetao Wang, Minfu Yang, Marcus Hacker, Haitao Zhou, Rui Yan, Sijin Li

**Affiliations:** 1 Department of Nuclear Medicine, First Hospital of Shanxi Medical University, No. 85 Jiefang South Road, Taiyuan, 030001 Shanxi China; 2 Division of Nuclear Medicine, Department of Biomedical Imaging and Image-guided Therapy, Medical University of Vienna, Vienna, Austria; 3 Collaborative Innovation Center for Molecular Imaging of Precision Medicine, Taiyuan, Shanxi China; 4 Department of Nuclear Medicine, The Third Affiliated Hospital of Soochow University, Changzhou, Jiangsu China; 5 Department of Nuclear Medicine, Beijing Chao-yang Hospital, Capital Medical University, Beijing, China; 6 Shanxi Key Laboratory of Molecular Imaging, Shanxi Medical University, Taiyuan, Shanxi China; 7 Key Laboratory of Cellular Physiology, Ministry of Education, Shanxi Medical University, Taiyuan, Shanxi China

**Keywords:** Microvascular dysfunction, PET, Myocardial blood flow

## Abstract

**Background:**

Coronary microvascular dysfunction (CMD) may precede clinically overt coronary artery disease (CAD). Overall and central obesity (CO) are major risk factors for CAD. This study sought to investigate the subclinical significance of body adiposity patterns based on the CMD risk.

**Methods:**

A total of 128 patients with non-obstructive CAD were prospectively enrolled. Patients were categorized into 4 anthropometric groups: normal weight and non-CO (NWNCO, *n* = 41), normal weight and CO (NWCO, *n* = 20), excess weight and non-CO (EWNCO, *n* = 26), and excess weight and CO (EWCO, *n* = 41). Patients underwent rest/stress electrocardiography-gated ^13^N-ammonia positron emission tomography to measure absolute myocardial blood flow (MBF), myocardial flow reserve (MFR), hemodynamic parameters, and cardiac function.

**Results:**

Resting MBF did not differ between groups (*P *= .36). Compared with the NWNCO group, hyperemic MBF and MFR were significantly lower in the NWCO and EWCO groups. Notably, patients with NWCO presented the lowest hyperemic MBF and MFR and the highest incidence of CMD. Waist circumference was an independent risk factor for CMD (OR 1.05, 95% CI 1.01 to 1.10, *P *= .02).

**Conclusion:**

In patients with non-obstructive CAD, CO may be associated with an increased risk of CMD to better fit the study findings which did not assess management or monitoring of MBF and MFR.

**Supplementary Information:**

The online version contains supplementary material available at 10.1007/s12350-021-02788-3.

## Introduction

Obesity is a global epidemic resulting in an increase in cardiovascular disease (CVD). Obesity can be divided into two types—overall obesity measured by body mass index (BMI) and central obesity (CO) measured by waist circumference (WC). CO has been associated with increased cardiometabolic risk and impaired cardiac function and is predictive of subclinical atherosclerosis and cardiovascular disease.^1^–^3^ CO is also strongly correlated with mental stress, inadequate sleep, and an unhealthy lifestyle,^4^ all of which are related to a higher prevalence of adverse cardiac events.^5^–^7^

Angina pectoris affects approximately 112 million people globally; however, a large proportion of these patients (up to 70%) do not present with obstructive coronary artery disease (CAD).^8^ Coronary microvascular dysfunction (CMD) occurs early in the progression of atherosclerosis among patients with non-obstructive CAD and may precede obstructive plaque formation and significant angiographic stenosis.^9^ Pioneering positron emission tomography (PET) studies have demonstrated an association between overall obesity and impairment of coronary circulatory or microvascular function.^10^–^12^ However, at present, there are no reports documenting the impact of different adiposity patterns in CMD.

Conventional non-invasive testing for cardiac risk assessment in CAD includes measuring the angiographic stenosis or obstruction severity and quantification of left ventricular function. However, these approaches do not effectively account for CMD, especially in subclinical high-risk subgroups. Electrocardiography (ECG)-gated myocardial perfusion imaging (MPI) with ^13^N-ammonia PET has demonstrated excellent diagnostic accuracy of CMD through evaluating myocardial blood flow (MBF) as well as myocardial flow reserve (MFR) and offers the added value of measuring hemodynamic parameters and cardiac function.^13^,^14^ In this study, we sought to investigate the associations between CMD and patterns of body adiposity based on BMI and WC among patients with non-obstructive CAD through ECG-gated ^13^N-ammonia PET-MPI.

## Methods

### Study Population and Design

Patients were prospectively enrolled in our study between March 2017 and January 2021. The study population included consecutive patients who underwent ECG-gated ^13^N-ammonia PET-MPI for evaluation of suspected CMD based on clinical symptoms, but not obstructive CAD (defined as ≥ 50% luminal stenosis) confirmed by clinically indicated invasive coronary arteriography or coronary computed tomography angiography within 3 months prior to the PET study. The most common indication for testing was the evaluation of new or worsening symptoms, including typical and atypical angina, dyspnea, or a decrease in general performance. Patients younger than 18 years, having coronary revascularization, or with contraindications against adenosine/dipyridamole or PET were excluded.^15^ Contraindications for the use of adenosine/dipyridamole were second- or third-degree atrioventricular block, hypotension, bradyarrhythmias, asthma, or hypersensitivity to dipyridamole/adenosine. This study was approved by the Ethics Review Committee of the First Hospital of Shanxi Medical University and a written informed consent was obtained from all patients prior to study participation.

Patients' clinical history, BMI, WC, and current medication use were ascertained at the time of PET imaging. Patients with a BMI < 25 kg/m^2^ were classified as having a normal weight, while those with a BMI ≥ 25 kg/m^2^ were categorized as having excess weight (i.e., overweight/obese).^16^ WC was measured by a trained examiner using a measuring tape positioned at the midpoint between the lower costal margin and iliac crest. The presence of CO was defined as WC > 95 cm for men or WC > 90 cm for women.^17^ Patients were subcategorized into the following four anthropometric groups: normal weight and non-CO (NWNCO), normal weight and CO (NWCO), excess weight and non-CO (EWNCO), and excess weight and CO (EWCO).

### ^13^N-Ammonia PET-MPI Protocol

A 1-day rest-stress protocol was used for ^13^N-ammonia PET-MPI. All patients were asked not to drink alcohol, tea, or coffee for 24 hours before each PET scan. Vasoactive medications or theophylline-containing medications were withheld for at least two half-lives prior to testing. All patients were examined in the fasting state. PET was performed using the GE Discovery VCT with 64-slice computed tomography (GE Healthcare, Waukesha, Wisconsin) in two-dimensional mode. A transmission scan was performed for attenuation correction before each PET acquisition. Myocardial perfusion was measured at rest and during maximal hyperemia by a standard intravenous infusion of adenosine or dipyridamole using ^13^N-ammonia as the flow tracer, as described previously.^13^,^18^ There was no statistically significant difference in the measured MPI or quantitative MBF when comparing the two agents in previous reports.^19^,^20^ Heart rate, blood pressure, and patients' electrocardiogram readings were recorded at baseline, throughout the infusion of the pharmacologic agents, and during recovery. Heart rate response (HRR) was defined as %HRR through the following equation (HRR, [(peak effect HR—baseline HR)/baseline HR] × 100).

### Data Reconstruction and Image Analysis

Images were reconstructed using a filtered back-projection algorithm and were resliced in short axis and in vertical and horizontal long-axis orientation. The summed stress score, summed rest score, and summed difference score were calculated (20-segment scoring); A scan was considered normal if the summed stress score was < 4.^13^ As previously reported,^18^,^21^,^22^ absolute myocardial perfusion was quantified through HeartSee software (University of Texas-Houston, Houston, Texas, FDA 150(k) K171303). The software used arterial inputs personalized for each PET from among five aortic and left atrium locations and automatically calculated absolute myocardial perfusion. MBF (mL/min/g) was analyzed for the global left ventricle. MFR was calculated as the ratio of stress to rest (absolute) MBF for the left ventricle. Hyperemic MBF < 2.3 mL/min/g or MFR < 2.5 was considered indicative of CMD according to previous studies.^23^–^26^ Left ventricle ejection fraction (LVEF) was automatically calculated through Myovation software (GE Healthcare, Xeleris) based on PET-gated data.

### Statistical Analysis

Data are presented as mean±SD or medians and interquartile ranges for continuous variables. Categorical variables are presented as frequencies and percentages. Differences between the four groups were assessed using one-way analysis of variance, Kruskal–Wallis test, chi-squared test, and Fisher's exact test as appropriate, with Bonferroni correction to account for multiple testing. Univariate and multivariate logistic regression analyses were performed to ascertain the factors associated with CMD. Values are expressed as odds ratio (OR) with 95% confidence interval (CI). No statistically significant interactions were found in the logistic regression models and no multicollinearity was present. A two-tailed *P *value < .05 was considered to be statistically significant. Statistical analyses were performed with IBM SPSS Version 22 (IBM Corporation, Armonk, NY, USA).

## Results

### Patient Characteristics

Table 1 summarizes the demographic and clinical characteristics of the 128 participating patients. Of these, 61 (47.66%) patients were categorized into the normal weight group (BMI < 25 kg/m^2^); 67 (52.34%) were categorized into the excess weight group (BMI ≥ 25 kg/m^2^), where 13 patients (10.16%) had a BMI ≥ 30 kg/m^2^. Of all patients, 61 (47.66%) were categorized into the centrally obese group. Notably, all patients with overall obesity also had CO. Thus, the patients with a BMI ≥ 30 kg/m^2^ were included in the EWCO group. An increased prevalence of hyperlipidemia was observed among the patients with CO. However, there were no significant differences in other cardiovascular risk factors (sex, diabetes, smoking, and family history of CAD) between the four groups.

**Table 1:** Demographic and clinical characteristics of the study patients

Characteristic	Total (*n* = 128)	NWNCO (*n* = 41)	NWCO (*n* = 20)	EWNCO (*n* = 26)	EWCO (*n* = 41)	*P *value
Males, *n* (%)	55 (43.0)	15 (36.6)	9 (45.0)	9 (34.6)	22 (53.7)	.349
Age (years)	54 ± 9	56 ± 9	57 ± 5	52 ± 8*	51 ± 9*^ϕ^	.028
BMI (kg/m^2^)	25.62 (23.58–27.51)	22.59 (20.95–24.01)	24.06 (23.46–24.43)	26.32 (25.63–26.82)*^ϕ^	28.05 (26.76–30.11)*^ϕ^	.001
WC (cm)	91 (84.25–97)	82 (78.88–86)	95 (90.25–96.75)*	88.5 (86–90.25)	98 (94.25–102)*^Γ^	.001
Major clinical symptoms, *n* (%)						
Typical angina	48 (37.5)	17 (41.5)	7 (35)	14 (53.8)	10 (24.4)	.098
Atypical angina	60 (46.9)	20 (48.8)	10 (50)	9 (34.6)	21 (51.2)	.569
Dyspnea	20 (15.6)	4 (9.8)	3 (15)	3 (11.5)	10 (24.4)	.284
Risk factors, *n* (%)						
Diabetes	40 (31.3)	9 (22)	7 (35)	7 (26.9)	17 (41.5)	.271
Hypertension	70 (54.7)	13 (31.7)	11 (55.0)	15 (57.7)*	31 (75.6)*	.001
Hyperlipidemia	73 (57.0)	15 (36.6)	15 (75.0)*	15 (60.0)	28 (68.3)*	.007
Smoking	44 (34.4)	12 (29.3)	10 (50)	7 (26.9)	15 (36.6)	.352
Family history of CAD	28 (21.9)	8 (19.5)	4 (20)	5 (19.2)	11 (26.8)	.86
Medications, *n* (%)						
Statin	50 (39.1)	8 (19.5)	11 (55.0)*	13 (50.0)*	18 (43.9)*	.015
Antiplatelet agent	27 (21.1)	2 (4.9)	7 (35.0)*	8 (30.8)*	10 (24.4)*	.013
Beta–blocker	22 (17.2)	7 (17.1)	3 (15.0)	4 (15.4)	8 (19.5)	.975
ACE inhibitor or ARB	31 (24.2)	7 (17.1)	7 (35.0)	5 (19.2)	12 (29.3)	.358
Calcium–channel blocker	35 (27.3)	5 (12.2)	6 (30.0)	7 (26.9)	17 (41.5)*	.03
Diuretic	4 (3.1)	0 (0)	0 (0)	1 (3.8)	3 (7.3)	.222
Fasting glucose values (mmol/L)	5.21 (4.74–5.93)	4.73 (4.5–5.43)	5.38 (4.81–7.38)	5.7 (4.99–6.71)	5.27 (4.97–6.27)	.107
Number of vessels diseased, *n* (%)						.155
0-vessel disease	60 (46.9)	21 (51.2)	9 (45)	16 (61.5)	14 (34.1)	
1-vessel disease	40 (31.3)	13 (31.7)	5 (25)	8 (30.8)	14 (34.1)	
2-vessel disease	14 (10.9)	4 (9.8)	1 (5)	1 (3.8)	8 (19.5)	
3-vessel disease	14 (10.9)	3 (7.3)	5 (25)	1 (3.8)	5 (12.2)	
Degree of coronary artery stenosis, no (%)						.707
0–24%	101 (78.9)	33 (80.5)	16 (80)	22 (84.6)	30 (73.2)	
25–49%	27 (21.1)	8 (19.5)	4 (20)	4 (15.4)	11 (26.8)	

Values are shown as *n* (%), mean ± SD or medians (interquartile ranges). The *P* values were obtained using the chi-squared test, ANOVA, or Kruskal–Wallis test, as appropriate

*Compared with NWNCO, *P*<0.05; ^ϕ^Compared with NWCO, *P* < .05; ^Γ^Compared with EWNCO, *P*<0.05

*NWNCO* normal weight and non-central obesity, *NWCO* normal weight and central obesity, *EWNCO* excess weight and non-central obesity, *EWCO* excess weight and central obesity, *BMI* body mass index, *WC* waist circumference, *CAD* coronary artery disease, *ACE* angiotensin-converting enzyme, *ARB* angiotensin receptor blocker

### Hemodynamic Parameters

At baseline, there was no significant difference with regard to heart rate and blood pressure among the four groups. Pharmacologic vasodilation produced significant increases in heart rate, which did not differ between the study groups. Although HRR was lower in patients with CO, these differences were not statistically significant. Blood pressure did not differ at the level of statistical significance among the four groups after pharmacologic vasodilation (Table 2).

**Table 2:** Patient hemodynamic and ECG-gated ^13^N-ammonia PET parameters

	NWNCO (*n* = 41)	NWCO (*n* = 20)	EWNCO (*n* = 26)	EWCO (*n* = 41)	*P* value
Heart rate (bpm)					
Baseline	64.54 ± 9.07	65.25 ± 7.52	63.85 ± 11.49	68.51 ± 10.90	.20
Peak effect	90.95 ± 13.01	86.85 ± 10.66	90.73 ± 8.36	92.44 ± 13.87	.42
HRR (%)	41.73 ± 15.72	33.54 ± 13.33	44.68 ± 17.88	37.20 ± 26.31	.21
SBP (mmHg)					
Baseline	126.68 ± 20.20	122.3 ± 16.09	135.54 ± 18.65	133.02 ± 19.87	.06
Peak effect	121.28 ± 16.28	125.35 ± 23.91	121.92 ± 13.91	126.22 ± 18.61	.59
DBP (mmHg)					
Baseline	70.20 ± 11.03	73.0 ± 11.18	75.46 ± 10.59	75.32 ± 9.83	.12
Peak effect	65.80 ± 10.72	66.15 ± 11.34	68.12 ± 9.63	70.0 ± 9.81	.28
LVEF (%)					
Rest	60.0 (54.25–64.75)	60.0 (57.00–66.00)	59.0 (54.50–62.00)	61.0 (50.25–65.75)	.74
Hyperemic	65.50 (57.75–69.75)	70.0 (64.0–72.0)	66.0 (62.0–69.0)	65.5 (59.75–68.75)	.17
LVEF reserve	5.5 (1.25–9.0)	6.0 (3.0–12.0)	7.0 (2.5–11.0)	4.0 (0–8.75)	.16
Summed score					
Summed stress score	4 (2.5–8)	4 (3–6)	6 (2.5–7.5)	4 (2–7)	.75
Summed rest score	1 (0–3)	0 (0–1)	1 (0–4)	1 (0–2)	.06
Summed difference score	2 (1–4.5)	3 (2–4)	3 (2–4.5)	3 (2-5)	.65
Baseline MBF (mL/min/g)	1.08 ± 0.3	1.01 ± 0.32	1.03 ± 0.37	0.96 ± 0.23	.36
Hyperemic MBF (mL/min/g)	3.52 ± 1.24	2.58 ± 0.76	3.1 ± 0.91	2.73 ± 0.91	.001
MFR	3.29 ± 0.8	2.7 ± 0.79	3.14 ± 0.77	2.88 ± 0.85	.03

Values are shown as mean ± SD or medians (interquartile ranges). The *P* values were obtained using the ANOVA or Kruskal–Wallis test, as appropriate

*Statistically significant difference between groups, *P* < .05

*NWNCO* normal weight and non-central obesity, *NWCO* normal weight and central obesity, *EWNCO* excess weight and non-central obesity, *EWCO* excess weight and central obesity, *SBP* systolic blood pressure, *DBP* diastolic blood pressure, *LVEF* left ventricle ejection fraction, *HRR* heart rate reserve, *MBF* myocardial blood flow, *MFR* myocardial flow reserve

### ECG-Gated ^13^N-Ammonia PET Results

At baseline, no statistically significant differences in LVEF were observed between the four study groups. Pharmacologic vasodilation produced statistically significant increases in LVEF, although hyperemic LVEF was comparable among the study groups. Consequently, no difference was observed in the adenosine/dipyridamole-induced LVEF changes (LVEF reserve, *P *= .16). There were no significant differences in the semiquantitative perfusion variables between the four groups (Table 2).

**Figure 1:**
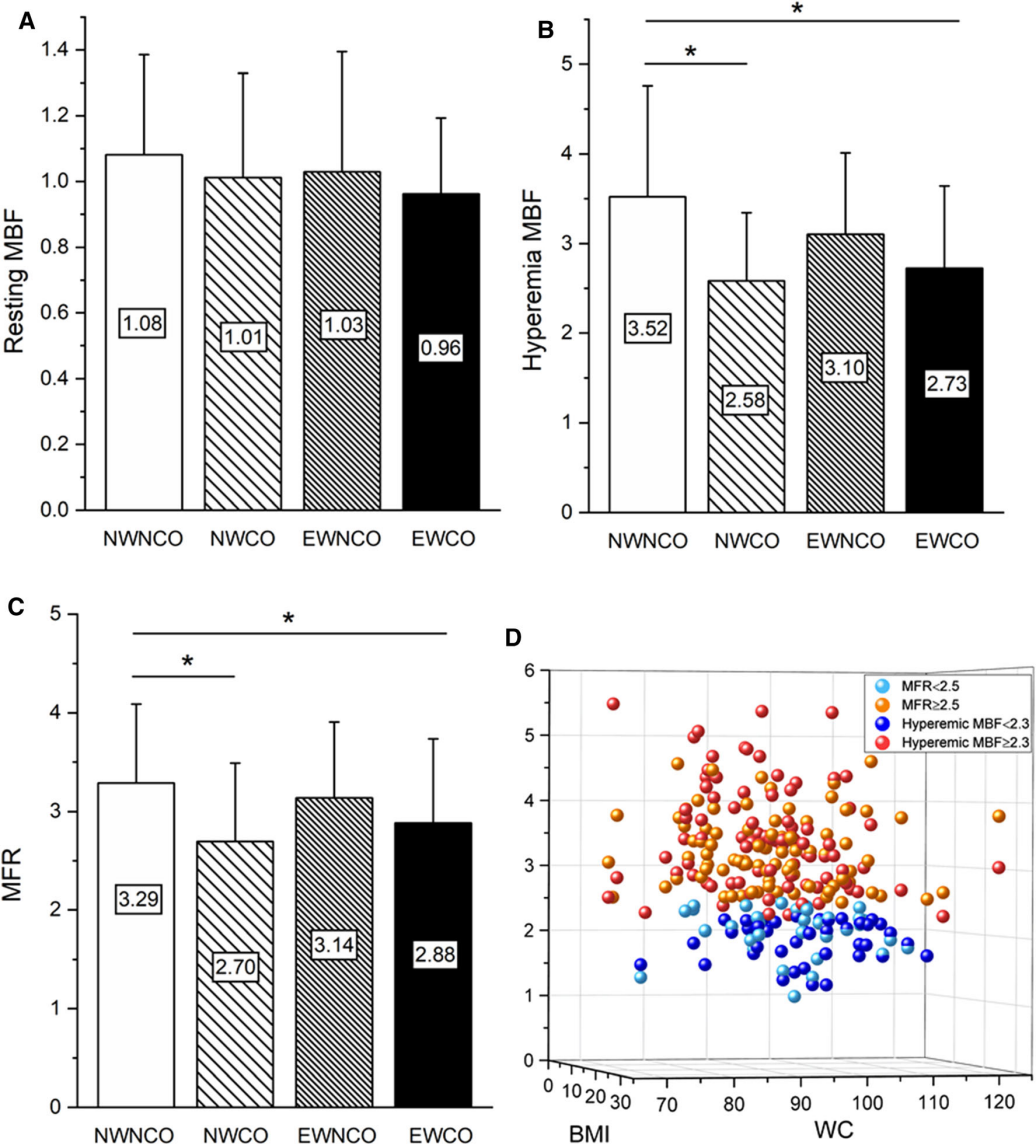
MBF and MFR. The figure presents MBF at rest (**A**) and during hyperemic flow stimulation (**B**), as well as the corresponding MFR (**C**) in the four study groups. Besides, the figure also presents the distributions of hyperemic MBF and MFR by both BMI and WC as continuous variables (**D**). Patients with CO had lower hyperemic MBF and MFR. Both hyperemic MBF and MFR were lowest in the NWCO group. *Compared with NWNCO, *P *< .05

**Figure 2:**
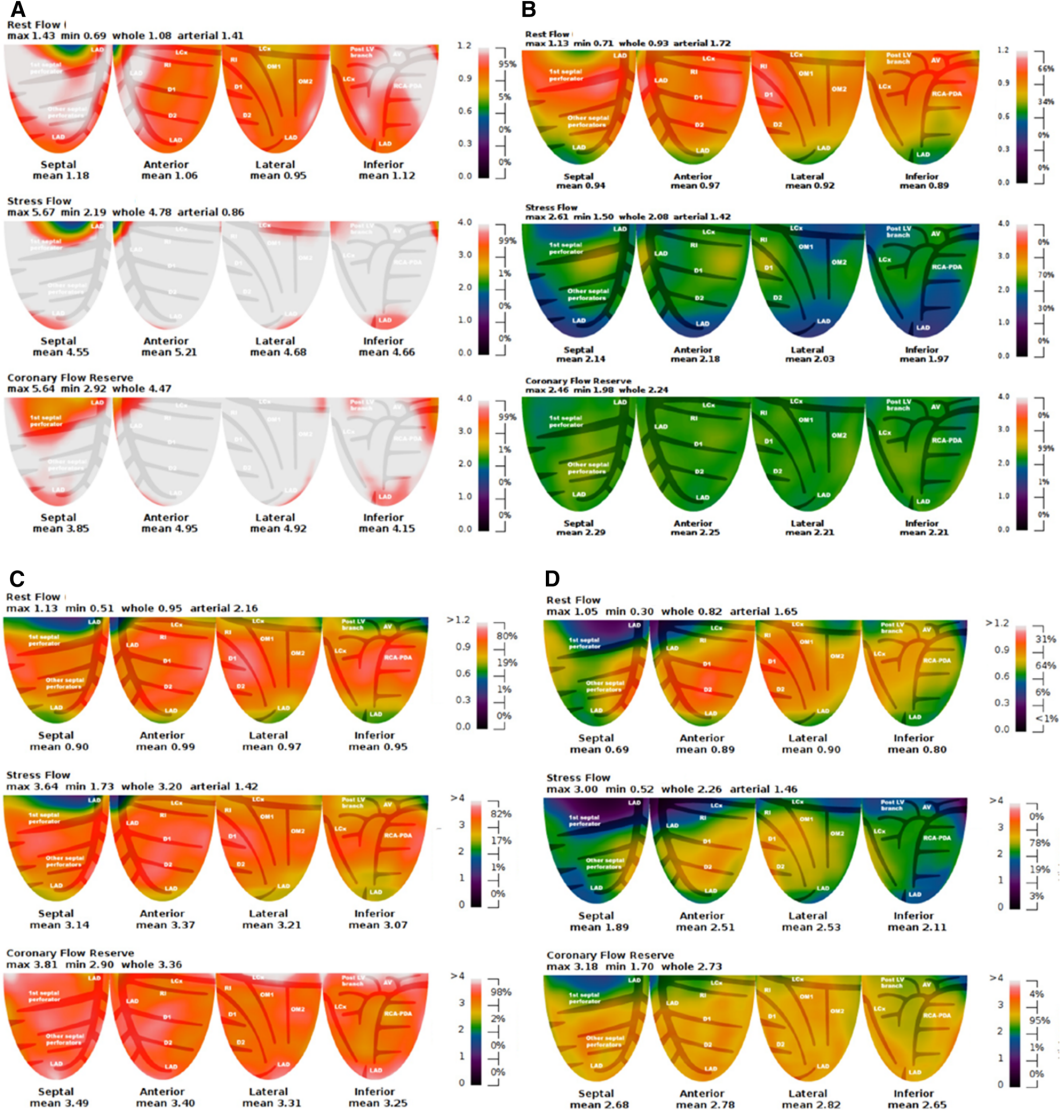
MBF and MFR in four typical cases representative of the different adiposity patterns. **A** A 53-year-old male patient with NWNCO, resting MBF = 1.08 mL/min/g, hyperemic MBF = 4.78 mL/min/g, and MFR = 4.47. **B** A 43-year-old male patient with NWCO, resting MBF =0.93 mL/min/g, hyperemic MBF = 2.08 mL/min/g, and MFR = 2.24. **C** A 55-year-old male patient with EWNCO, resting MBF = 0.95 mL/min/g, hyperemic MBF = 3.20 mL/min/g, and MFR = 3.36. **D** A 55-year-old male patient with EWCO, resting MBF = 0.82 mL/min/g, hyperemic MBF = 2.26 mL/min/g, and MFR = 2.73

The mean global MBF and MFR of the four study groups are presented in Table 2 and Figure 1. The proportion of adenosine as a pharmacologic agent was 29.3%, 35%, 26.9%, and 19.5% in the NWNCO, NWCO, EWNCO, and EWCO groups, respectively; there were no significant differences between the four groups (*P *= .587). At baseline, MBF did not differ among the study groups (*P *= .36). Compared with the NWNCO group, hyperemic MBF was significantly lower in the CO groups (NWCO vs NWNCO, *P *= .001; EWCO vs NWNCO, *P *= .001). In patients with excess weight, hyperemic MBF was somewhat lower in patients with CO, but this difference was not statistically significant (*P *= .14). Hyperemic MBF was comparable among the groups of patients with CO (*P *= .60). Similarly, no statistically significant differences were found between the groups without CO (*P *= .10). Notably, hyperemic MBF was lowest in the NWCO group (2.58 ± 0.76 mL/min/g). MFR was significantly lower in the groups with CO compared to the NWNCO group (NWCO vs NWNCO, *P *= .01; EWCO vs NWNCO, *P *= .03). In patients with excess weight, MFR was somewhat lower in patients with CO, but this difference was not statistically significant (*P *= .21). MFR did not differ between the groups with CO (*P *= .40) or without CO (*P *= .47). Likewise, MFR was lowest in the NWCO group (2.70 ± 0.79). Figure 2 shows the MBF and MFR of representative cases in the NWNCO, NWCO, EWNCO, and EWCO groups with non-obstructive CAD.

In patients with CO (*n* = 13) presenting a BMI ≥ 30 kg/m^2^, the mean global resting MBF was 0.91 ± 0.16 mL/min/g, the mean hyperemic MBF was 2.60 ± 0.75 mL/min/g, and the mean MFR was 2.90 ± 0.88.

### Correlation of Risk Factors with CMD

**Figure 3:**
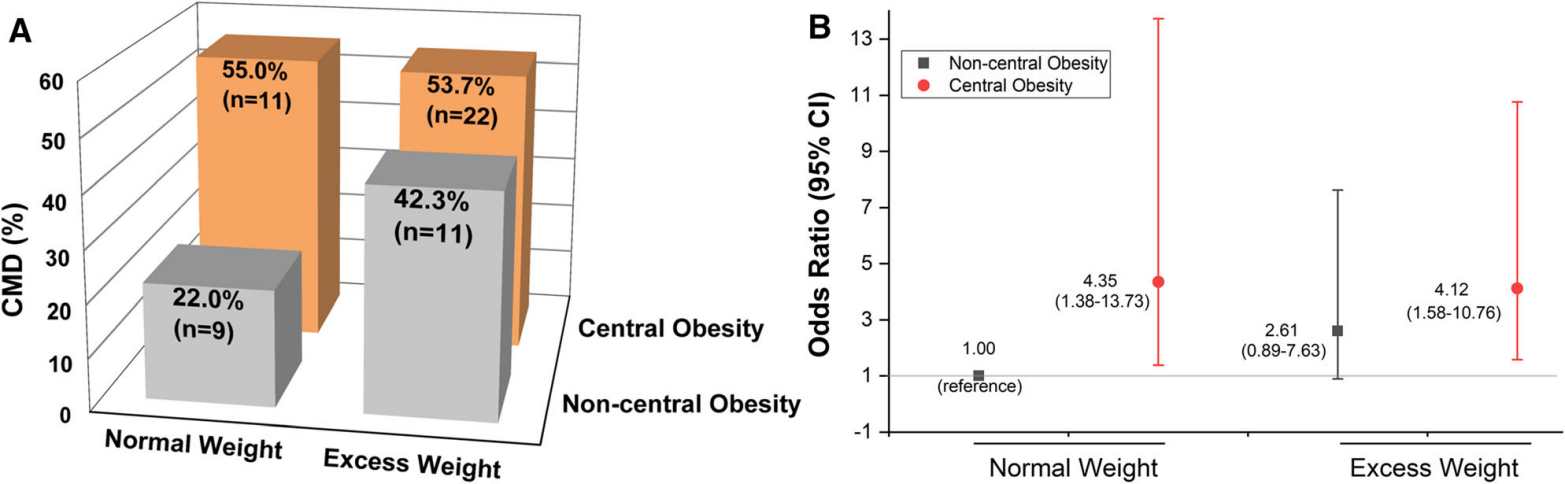
Effect of different adiposity patterns on CMD. The figure presents the crude prevalence rate of CMD in the four groups (**A**) as well as the connection of different adiposity patterns with CMD risk (**B**). Patients with NWCO had the highest prevalence and risk of CMD

We observed statistically significant differences with regard to prevalence rate of CMD in patients with different adiposity patterns (*P *= .015, Figure 3a). The crude prevalence rate of CMD was maximal in patients with NWCO (*n* = 11, 55%) and minimal among patients with NWNCO (*n* = 9, 22%). In patients with excess weight, 43.2% of patients without CO (*n* = 11) and 53.7% of patients with CO (*n *= 22) presented with CMD. In the EWCO group, the initial assessment identified 7 out of 13 (53.8%) patients with obesity having CMD. Correspondingly, compared with the NWNCO group, NWCO (OR = 4.35, 95% CI 1.38 to 13.73, *P *= .01) and EWCO (OR = 4.12, 95% CI 1.58 to 10.76, *P *= .004) were significantly associated with a higher risk of CMD within univariate analyses (Figure 3b).

**Figure 4:**
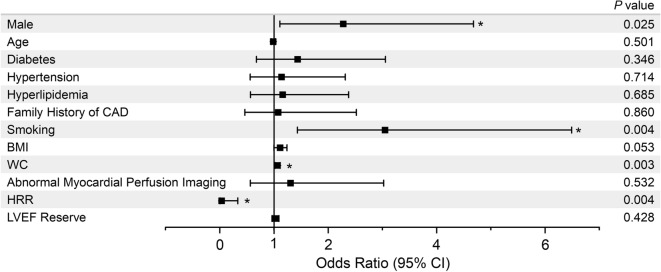
Predictors of CMD in univariate regression analysis

The impact of potential risk factors on the risk of CMD was evaluated across subgroups through univariate analyses (Figure 4). Univariate regression analysis identified male sex, smoking, WC, and blunted HRR as significant predictors of CMD. Factors with *P *< .1 in univariate analyses were included in the multivariate analysis models. After multivariate adjustment, smoking (OR = 2.30, 95% CI 1.03 to 5.13, *P *= .04) and WC (OR = 1.05, 95% CI 1.01 to 1.10, *P *= .02) were independent risk factors for CMD and HRR was an independent protective factor with regard to CMD risk (OR = 0.08, 95% CI 0.01 to 0.84, *P *= .04, Table 3). The Hosmer–Lemeshow test of model fit showed a *P *value of .766. The area under the receiver operating characteristic curve was 0.74 (95% CI 0.653 to 0.826).

**Table 3:** Independent predictors of CMD through multivariate analysis with forward selection

Variables	OR	95% CI	*P *value
Smoking	2.30	1.03–5.13	.04
WC	1.05	1.01–1.10	.02
HRR	0.08	0.01–0.84	.04

Regression analysis was performed accounting for sex, smoking, BMI, WC, and HRR. Only the variables retained in the final model are shown here

*CMD* coronary microvascular dysfunction, *OR* odds ratio, *CI* confidence interval, *WC* waist circumference, *HRR* heart rate reserve

## Discussion

In this study, we investigated the integrated predictive value of two independent anthropometric indices (BMI and WC) on CMD risk in patients with non-obstructive CAD. We demonstrated, to our knowledge, for the first time that patients with CO showed lower hyperemic MBF and MFR, as well as a higher prevalence of CMD. In particular, patients with NWCO presented the lowest hyperemic MBF and MFR and the highest incidence rates of CMD among patients with non-obstructive CAD. Furthermore, we demonstrated that being overweight or obese based on BMI criteria does not lead to a higher risk of CMD in the absence of CO. Thus, measurement of WC may complement the use of BMI in CMD risk stratification, especially for patients with a normal BMI.

We present several explanations for our findings. First, patients with NWCO may have visceral fat accumulation and less muscle mass. Increased visceral adiposity has been associated with the development and progression of atherosclerotic disease.^11^ Insufficient muscle mass in a low BMI population would lead to adverse health outcomes since muscle mass is associated with a more favorable metabolic profile.^27^ Second, patients with NWCO may have decreased subcutaneous fat on their hips and legs. Gluteofemoral fat possesses an atheroprotective effect and its elevation is considered as a protective factor for CMD.^11^ A previous study found that CO was associated with higher systemic vascular resistance, whereas peripheral obesity showed the opposite association.^28^ Thus, CO may also be associated with high coronary microvascular resistance, a proxy for CMD.^8^

Presenting results were in line with a pioneering study using magnetic resonance imaging that showed that visceral adiposity was a superior predictor than BMI of reduced perfusion in women, suggesting that visceral fat may contribute to CMD.^29^ A recent transthoracic echocardiography study demonstrated that overall and central obesity were associated with different patterns of left ventricular structural and functional alterations, stressing the importance of incorporating BMI and WC measurements into assessing obesity-related left ventricular alterations.^30^

CO plays a crucial role in metabolic syndrome. Approximately 35% of the obese population are metabolically healthy obese, showing lower visceral adipose tissue and a predominantly lower body fat deposition.^31^ These individuals have about half the risk of developing CVD than metabolically unhealthy individuals with obesity, but they still have a higher risk than metabolically healthy individuals without obesity.^31^ Interestingly, it seems that individuals with normal weight are somewhat at higher risk of CVD associated with the metabolic dysfunction than individuals with obesity.^31^

Furthermore, chronic mental stress has been associated with the accumulation of abdominal adiposity in previous studies,^4^,^32^,^33^ and severe psychological stressors could contribute to diverse pathophysiological changes in CVD.^33^ Pioneering studies show that mental stress has increased dramatically over the past half century and that this stress may promote energy intake as well as preferential abdominal fat deposition through long-term activation of the hypothalamic–pituitary–adrenal (HPA) axis.^4^ Stress could also increase central sympathetic nervous system activity, which can contribute to endothelial dysfunction, a key pathological mechanism for CMD.^5^ The amygdala is a key component of the brain's salience network involved in stress pathways.^5^ Increasing neuropeptide Y neuron expression in the central amygdala can result in exaggerated obesity,^34^ and elevations of resting glucose metabolism in the amygdala could independently predict the development of cardiovascular disease.^5^

Poor sleep may also explain certain associations observed in this study. Inadequate sleep is extremely common in the general population, and it may contribute to the existing high prevalence of CO.^35^,^36^ Poor sleep could be seen as a stressor itself because lack of sleep increases the HPA-axis activation as a function of mental stress.^4^,^37^ In addition, inadequate sleep promotes low-level systemic inflammation that could in turn promote the development of CMD and atherosclerotic plaque formation.^38^ CO is also strongly correlated with an unhealthy lifestyle: poor diet, smoking, alcohol intake, and lack of physical activity.^4^ These lifestyle factors are independent risk factors for cardiovascular disease and may lead to CMD. Considered collectively, patients with CO, especially those with a normal BMI, may have an increased risk of CMD. Moreover, this could partly explain the previous study results that showed the association of NWCO with the highest risk of mortality among patients with CAD.^39^

ECG-gated 13N-ammonia PET-MPI provides the added value of information on hemodynamic parameters and cardiac function. Our study found that HRR was a protective factor for CMD, which was consistent with previous findings.^14^ Moreover, blunted HRR can provide incremental prognostic value for long-term cardiovascular outcomes.^40^ HRR may be taken into consideration for risk stratification in obese patients with non-obstructive CAD.

LVEF also had diagnostic and prognostic utility.^13^ Notably, supra-normal LVEF was associated with an increased risk of major adverse cardiac events, and CMD may account for this association.^13^ However, we found that LVEF was not a powerful predictor of CMD. We found that abnormal myocardial perfusion was also not a powerful predictor of CMD, which was in keeping with previous research.^14^ Although the traditional CAD risk factors of hypertension, hyperlipidemia, and diabetes may all contribute to the pathology of CMD, the association between these factors is not well established.^8^ In our study, we could not conclude that CMD was correlated with traditional CAD risk factors, with the exception of smoking. We found that smoking, which could lead to endothelial dysfunction, was strongly associated with CMD, consistent with the previous findings.^8^,^41^

## Limitations

Our study had certain limitations. First, since this was a single-center study, the findings may not be generalizable to a broad population. Second, statistical power could be limited owning to the relatively small sample size of this study. Furthermore, we inadvertently recruited an unbalanced subgroup based on the representative distribution of participants of normal weight or excess weight. Third, we did not collect information on potentially confounding health behaviors such as disordered eating and lack of exercise. We also did not measure the patients' body fat percentage, lean body mass, or fat distribution. Fourth, we justified the cut-off for CMD based on ^15^O–H_2_O rather than ^13^N-ammonia. Although ^13^N-ammonia and ^15^O-water provide similar absolute MBF information over a wide range of blood flows in human, it remains uncertain how these thresholds for hyperemic flows using ^15^O–H_2_O compare with ^13^N-ammonia. Moreover, we did not perform follow-up surveillance for future cardiovascular events.

## Conclusion

In this study, we report that CO may be associated with decreased coronary microvascular function in patients with non-obstructive CAD, with the highest level of CMD risk observed among patients with NWCO. Hence, hyperemic MBF and MFR could facilitate the clinical management of non-obstructive CAD patients. Future studies are warranted to investigate the potential role of MBF or MFR regarding CVD risk management, while considering a variety of body fat indices.

## New Knowledge Gained

In patients with non-obstructive CAD, patients with CO, especially those with normal weight, are more likely to have CMD. On the contrary, being overweight or obese based on BMI criteria does not lead to a higher risk of CMD in the absence of CO. Thus, measurement of WC may complement the use of BMI in CMD risk stratification, especially for patients with a normal BMI.

## Supplementary Information

Below is the link to the electronic supplementary material.

Supplementary file1 (PPTX 847 kb)

## Abbreviations

BMI: Body mass index
CAD: Coronary artery disease
CMD: Coronary microvascular dysfunction
CO: Central obesity
MBF: Myocardial blood flow
MFR: Myocardial flow reserve
MPI: Myocardial perfusion imaging
PET: Positron emission tomography
WC: Waist circumference

## References

[1] Kim S, Kyung C, Park JS, Lee SP, Kim HK, Ahn CW (2015). Normal-weight obesity is associated with increased risk of subclinical atherosclerosis. Cardiovasc Diabetol.

[2] He X, Liu C, Chen Y, He J, Dong Y (2018). Overweight without central obesity, cardiovascular risk, and all-cause mortality. Mayo Clin Proc.

[3] Bell JA, Carslake D, O'Keeffe LM, Frysz M, Howe LD, Hamer M (2018). Associations of body mass and fat indexes with cardiometabolic traits. J Am Coll Cardiol.

[4] Geiker NRW, Astrup A, Hjorth MF, Sjödin A, Pijls L, Markus CR (2018). Does stress influence sleep patterns, food intake, weight gain, abdominal obesity and weight loss interventions and vice versa?. Obes Rev.

[5] Tawakol A, Ishai A, Takx RA, Figueroa AL, Ali A, Kaiser Y (2017). Relation between resting amygdalar activity and cardiovascular events: A longitudinal and cohort study. Lancet.

[6] Franklin BA (2014). Preventing exercise-related cardiovascular events: Is a medical examination more urgent for physical activity or inactivity?. Circulation.

[7] McAlpine CS, Swirski FK (2016). Circadian influence on metabolism and inflammation in atherosclerosis. Circ Res.

[8] Kunadian V, Chieffo A, Camici PG, Berry C, Escaned J, Maas AHEM (2020). An eapci expert consensus document on ischaemia with non-obstructive coronary arteries in collaboration with european society of cardiology working group on coronary pathophysiology & microcirculation endorsed by coronary vasomotor disorders international study group. Eur Heart J.

[9] Safdar B, D'Onofrio G, Dziura J, Russell RR, Johnson C, Sinusas AJ (2020). Prevalence and characteristics of coronary microvascular dysfunction among chest pain patients in the emergency department. Eur Heart J-Acute Ca.

[10] Bajaj NS, Osborne MT, Gupta A, Tavakkoli A, Bravo PE, Vita T (2018). Coronary microvascular dysfunction and cardiovascular risk in obese patients. J Am Coll Cardiol.

[11] Valenta I, Dilsizian V, Quercioli A, Jüngling FD, Ambrosio G, Wahl R (2014). Impact of obesity and bariatric surgery on metabolism and coronary circulatory function. Curr Cardiol Rep.

[12] Quercioli A, Pataky Z, Vincenti G, Makoundou V, Di Marzo V, Montecucco F (2011). Elevated endocannabinoid plasma levels are associated with coronary circulatory dysfunction in obesity. Eur Heart J.

[13] Maredziak M, Bengs S, Portmann A, Haider A, Wijnen WJ, Warnock GI (2020). Microvascular dysfunction and sympathetic hyperactivity in women with supra-normal left ventricular ejection fraction (snLVEF). Eur J Nucl Med Mol.

[14] Haider A, Bengs S, Maredziak M, Messerli M, Fiechter M, Giannopoulos AA (2019). Heart rate reserve during pharmacological stress is a significant negative predictor of impaired coronary flow reserve in women. Eur J Nucl Med Mol.

[15] Bakula A, Patriki D, von Felten E, Benetos G, Sustar A, Benz D et al. Splenic switch-off as a novel marker for adenosine response in nitrogen-13 ammonia PET myocardial perfusion imaging: Cross-validation against CMR using a hybrid PET/MR device. J Nucl Cardiol 2020. 10.1007/s12350-020-02448-y PMC9163112 33354759

[16] Obesity: preventing and managing the global epidemic. Report of a WHO consultation. WHO Tech Rep Ser 2000;894:1-12 11234459

[17] Wu Y, Huxley R, Li L, Anna V, Xie G, Yao C (2008). Prevalence, awareness, treatment, and control of hypertension in China: Data from the China National Nutrition and Health Survey 2002. Circulation.

[18] Vasquez AF, Johnson NP, Gould KL (2013). Variation in quantitative myocardial perfusion due to arterial input selection. JACC-Cardiovasc Img.

[19] Bajaj NS, Singh A, Zhou W, Gupta A, Fujikura K, Byrne C (2020). Coronary microvascular dysfunction, left ventricular remodeling, and clinical outcomes in patients with chronic kidney impairment. Circulation.

[20] Chareonthaitawee P, Kaufmann PA, Rimoldi O, Camici PG (2001). Heterogeneity of resting and hyperemic myocardial blood flow in healthy humans. Cardiovasc Res.

[21] Kitkungvan D, Bui L, Johnson NP, Patel MB, Roby AE, Vejpongsa P (2019). Quantitative myocardial perfusion positron emission tomography and caffeine revisited with new insights on major adverse cardiovascular events and coronary flow capacity. Eur Heart J-Card Img.

[22] Kitkungvan D, Johnson NP, Roby AE, Patel MB, Kirkeeide R, Gould KL (2017). Routine clinical quantitative rest stress myocardial perfusion for managing coronary artery disease: Clinical relevance of test-retest variability. JACC-Cardiovasc Img.

[23] Danad I, Uusitalo V, Kero T, Saraste A, Raijmakers PG, Lammertsma AA (2014). Quantitative assessment of myocardial perfusion in the detection of significant coronary artery disease: cutoff values and diagnostic accuracy of quantitative [(15)O]H2O PET imaging. J Am Coll Cardiol.

[24] Promteangtrong C, Jantarato A, Kunawudhi A, Kiatkittikul P, Siripongsatian D, Boonkawin N et al. Clinical impact of quantitative [O] HO PET/CT myocardial perfusion imaging on decision-making regarding invasive management of coronary artery disease. J Nucl Cardiol 2021. 10.1007/s12350-021-02604-y 33826128

[25] Amigues I, Russo C, Giles J, Tugcu A, Weinberg R, Bokhari S (2019). Myocardial microvascular dysfunction in rheumatoid arthritis. Circ-Cardiocasc Imag.

[26] Graf S, Khorsand A, Gwechenberger M, Novotny C, Kletter K, Sochor H (2007). Typical chest pain and normal coronary angiogram: Cardiac risk factor analysis versus PET for detection of microvascular disease. J Nucl Med.

[27] Sun Y, Liu B, Snetselaar LG, Wallace RB, Caan BJ, Rohan TE (2019). Association of normal-weight central obesity with all-cause and cause-specific mortality among postmenopausal women. JAMA Netw Open.

[28] Neeland IJ, Gupta S, Ayers CR, Turer AT, Rame JE, Das SR (2013). Relation of regional fat distribution to left ventricular structure and function. Circ-Cardiovasc Imag.

[29] Hall M, Brinkley T, Chughtai H, Morgan T, Hamilton C, Jordan J (2016). Adiposity is associated with gender-specific reductions in left ventricular myocardial perfusion during Dobutamine Stress. PLoS ONE.

[30] Cai A, Liu L, Zhou D, Zhou Y, Tang S, Feng Y. The patterns of left ventricular alteration by adipose tissue distribution: implication for heart failure prevention. ESC Heart Failure 2021. 10.1002/ehf2.13415 PMC8318514 34037322

[31] Faidon M (2019). Metabolically healthy obesity: what's in a name?. Am J Clin Nutr.

[32] Björntorp P (2001). Do stress reactions cause abdominal obesity and comorbidities?. Obes Rev.

[33] Cizza G (2011). Major depressive disorder is a risk factor for low bone mass, central obesity, and other medical conditions. Dialogues Clin Neuro.

[34] Ip CK, Zhang L, Farzi A, Qi Y, Clarke I, Reed F (2019). Amygdala NPY circuits promote the development of accelerated obesity under chronic stress conditions. Cell Metab.

[35] Chaput JP, Després JP, Bouchard C, Tremblay A (2011). The association between short sleep duration and weight gain is dependent on disinhibited eating behavior in adults. Sleep.

[36] Chaput JP, Després JP, Bouchard C, Tremblay A (2012). Longer sleep duration associates with lower adiposity gain in adult short sleepers. Int J Obesity.

[37] Guyon A, Balbo M, Morselli LL, Tasali E, Leproult R, L'Hermite-Balériaux M (2014). Adverse effects of two nights of sleep restriction on the hypothalamic-pituitary-adrenal axis in healthy men. J Clin Endocr Metab.

[38] Faraut B, Boudjeltia KZ, Vanhamme L, Kerkhofs M (2012). Immune, inflammatory and cardiovascular consequences of sleep restriction and recovery. Sleep Med Rev.

[39] Coutinho T, Goel K, Corrêa de Sá D, Carter RE, Hodge DO, Kragelund C (2013). Combining body mass index with measures of central obesity in the assessment of mortality in subjects with coronary disease: Role of "normal weight central obesity. J Am Coll Cardiol.

[40] Gebhard CE, Marędziak M, Portmann A, Bengs S, Haider A, Fiechter M (2019). Heart rate reserve is a long-term risk predictor in women undergoing myocardial perfusion imaging. Eur J Nucl Med Mol.

[41] Feher A, Sinusas AJ (2017). Quantitative assessment of coronary microvascular function: dynamic single-photon emission computed tomography, positron emission tomography, ultrasound, computed tomography, and magnetic resonance imaging. Circ-Cardiocasc Imag.

