# Supplementary material for: Combining body mass index with waist circumference to assess coronary microvascular function in patients with non-obstructive coronary artery disease
Source: J Nucl Cardiol. 2021 Sep 3;29(5):2434–45. doi: 10.1007/s12350-021-02788-3 (PMC9553765; doi:10.1007/s12350-021-02788-3)
Supplement: Supplementary file 1 — Supplementary file1 (PPTX 847 kb) [file 12350_2021_2788_MOESM1_ESM.pptx]

## Slide 1
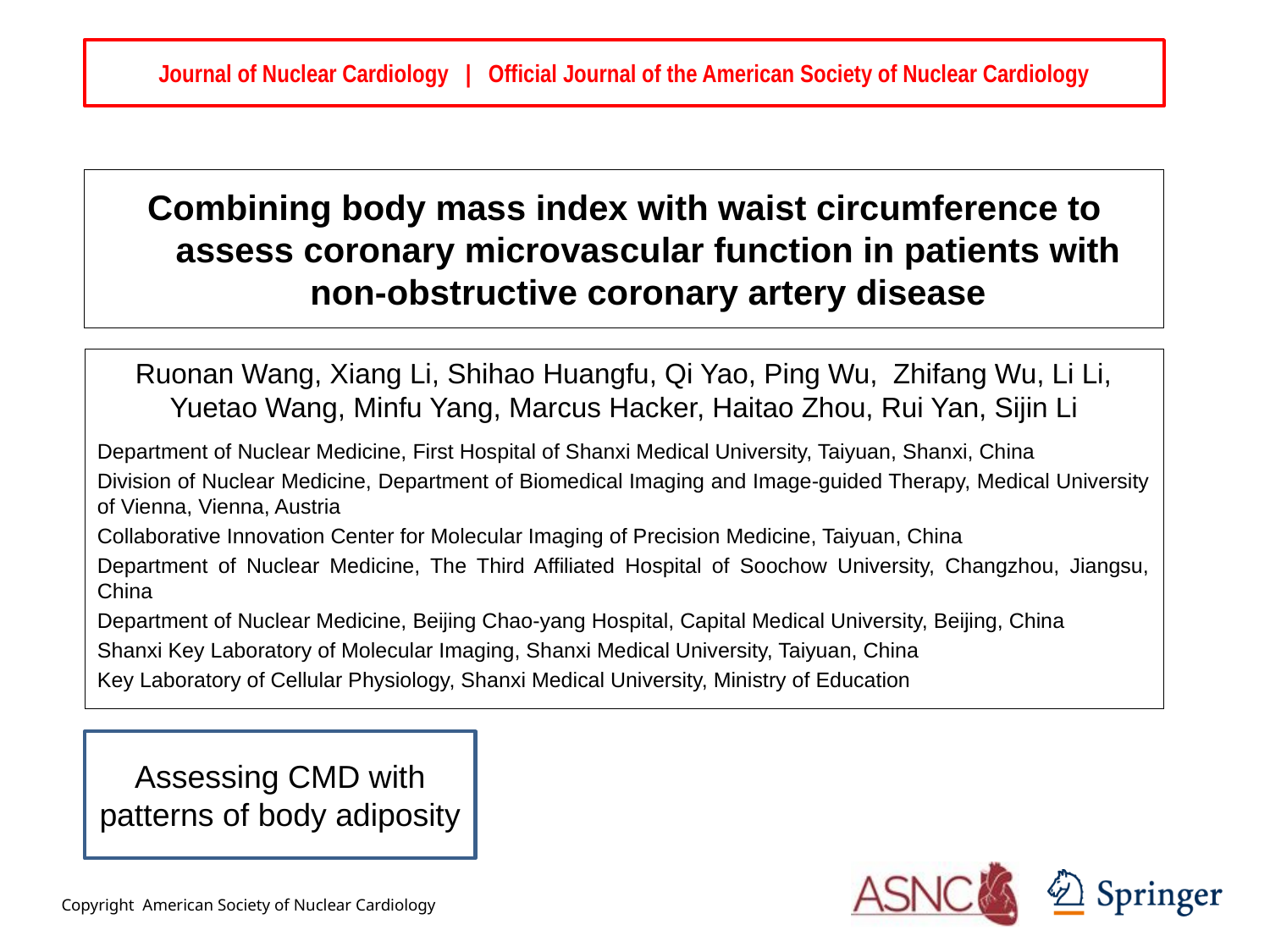

Journal of Nuclear Cardiology | Official Journal of the American Society of Nuclear Cardiology
# Combining body mass index with waist circumference to assess coronary microvascular function in patients with non-obstructive coronary artery disease
Ruonan Wang, Xiang Li, Shihao Huangfu, Qi Yao, Ping Wu, Zhifang Wu, Li Li, Yuetao Wang, Minfu Yang, Marcus Hacker, Haitao Zhou, Rui Yan, Sijin Li
Department of Nuclear Medicine, First Hospital of Shanxi Medical University, Taiyuan, Shanxi, China
Division of Nuclear Medicine, Department of Biomedical Imaging and Image-guided Therapy, Medical University of Vienna, Vienna, Austria
Collaborative Innovation Center for Molecular Imaging of Precision Medicine, Taiyuan, China
Department of Nuclear Medicine, The Third Affiliated Hospital of Soochow University, Changzhou, Jiangsu, China
Department of Nuclear Medicine, Beijing Chao-yang Hospital, Capital Medical University, Beijing, China
Shanxi Key Laboratory of Molecular Imaging, Shanxi Medical University, Taiyuan, China
Key Laboratory of Cellular Physiology, Shanxi Medical University, Ministry of Education
Assessing CMD with patterns of body adiposity
Copyright American Society of Nuclear Cardiology

## Slide 2
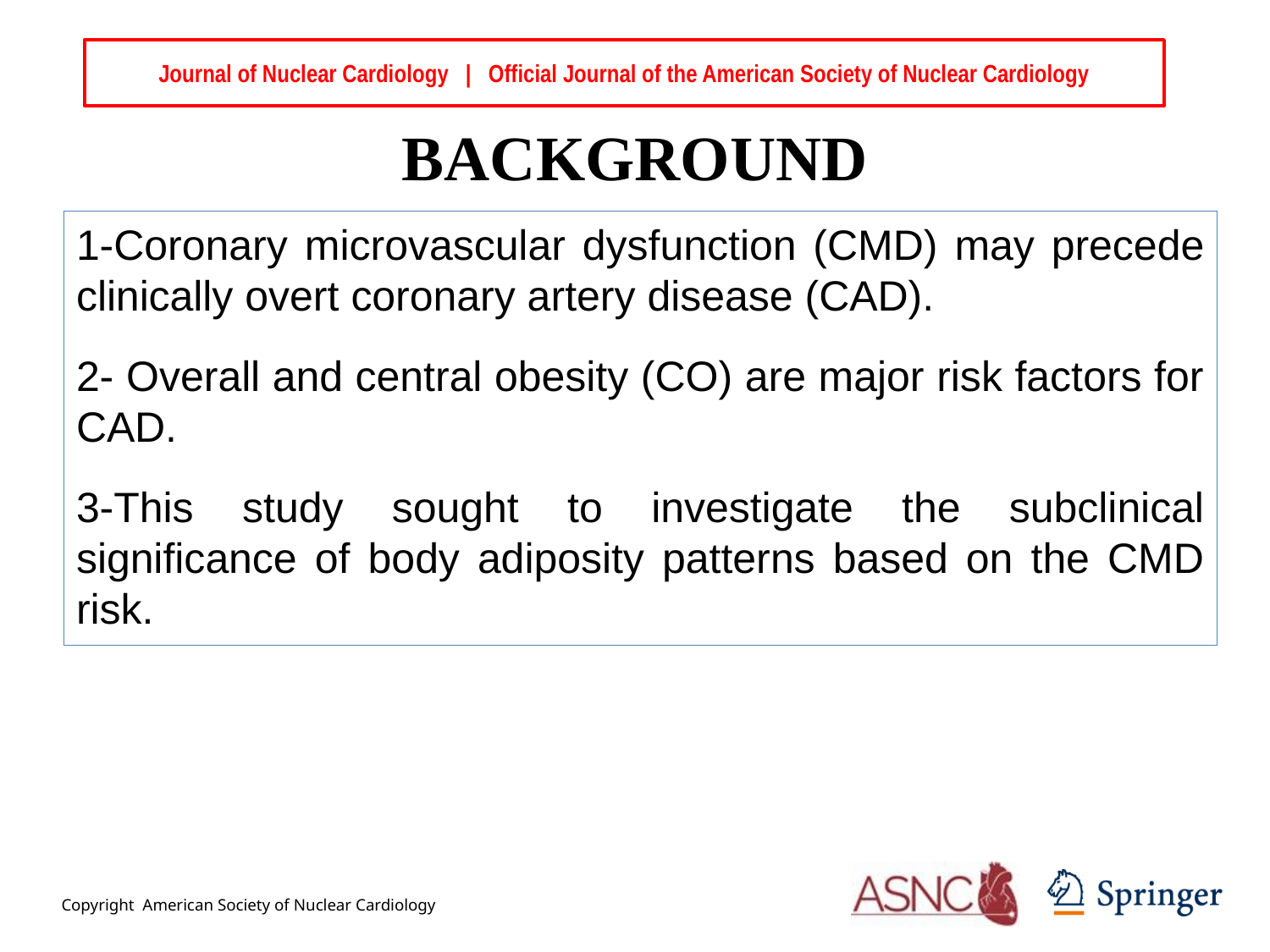

Journal of Nuclear Cardiology | Official Journal of the American Society of Nuclear Cardiology
# BACKGROUND
1-Coronary microvascular dysfunction (CMD) may precede clinically overt coronary artery disease (CAD).
2- Overall and central obesity (CO) are major risk factors for CAD.
3-This study sought to investigate the subclinical significance of body adiposity patterns based on the CMD risk.
Copyright American Society of Nuclear Cardiology

## Slide 3
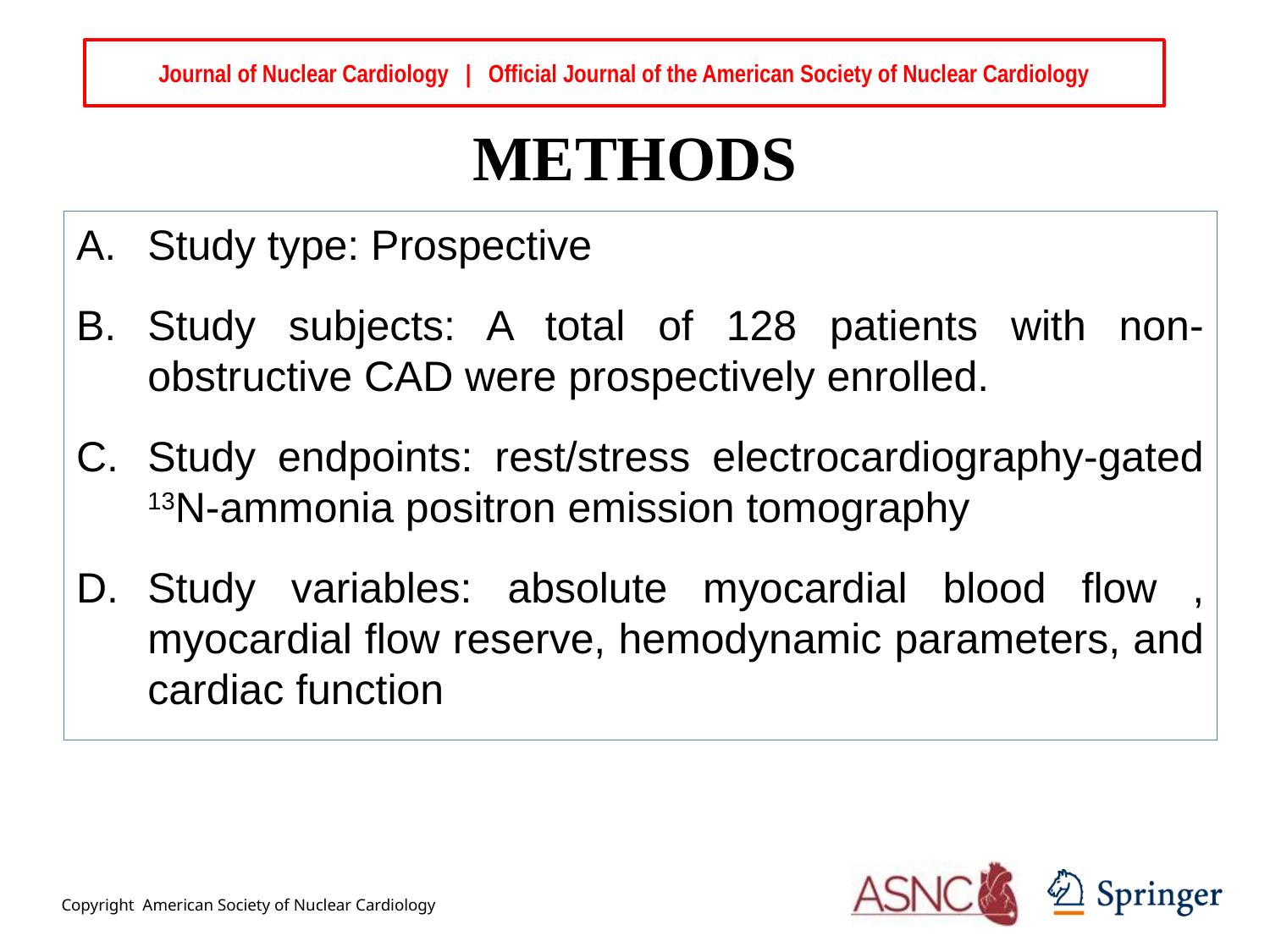

Journal of Nuclear Cardiology | Official Journal of the American Society of Nuclear Cardiology
# METHODS
Study type: Prospective
Study subjects: A total of 128 patients with non-obstructive CAD were prospectively enrolled.
Study endpoints: rest/stress electrocardiography-gated 13N-ammonia positron emission tomography
Study variables: absolute myocardial blood flow , myocardial flow reserve, hemodynamic parameters, and cardiac function
Copyright American Society of Nuclear Cardiology

## Slide 4
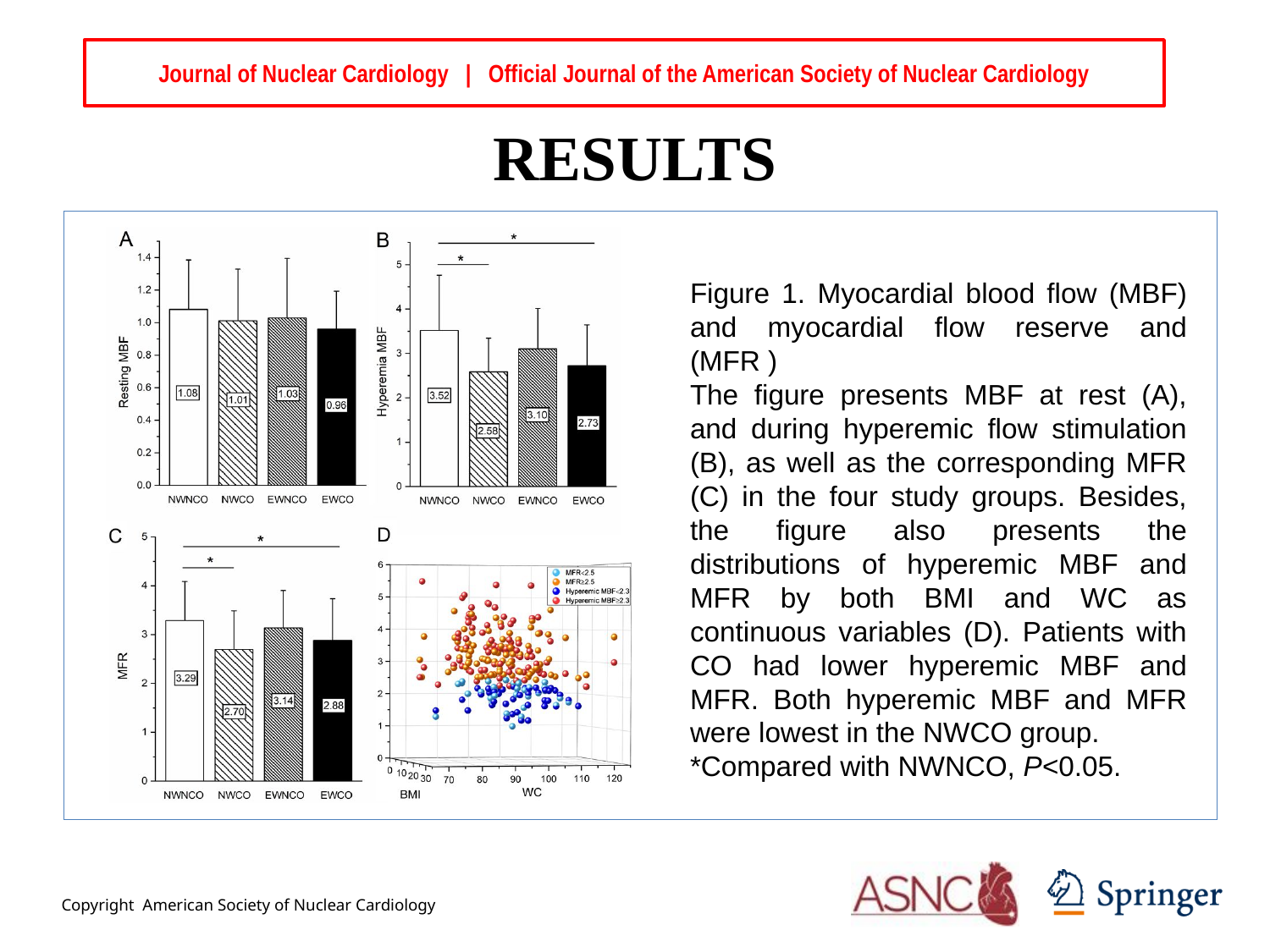

Journal of Nuclear Cardiology | Official Journal of the American Society of Nuclear Cardiology
# RESULTS
Figure 1. Myocardial blood flow (MBF) and myocardial flow reserve and (MFR )
The figure presents MBF at rest (A), and during hyperemic flow stimulation (B), as well as the corresponding MFR (C) in the four study groups. Besides, the figure also presents the distributions of hyperemic MBF and MFR by both BMI and WC as continuous variables (D). Patients with CO had lower hyperemic MBF and MFR. Both hyperemic MBF and MFR were lowest in the NWCO group.
*Compared with NWNCO, P<0.05.
Copyright American Society of Nuclear Cardiology

## Slide 5
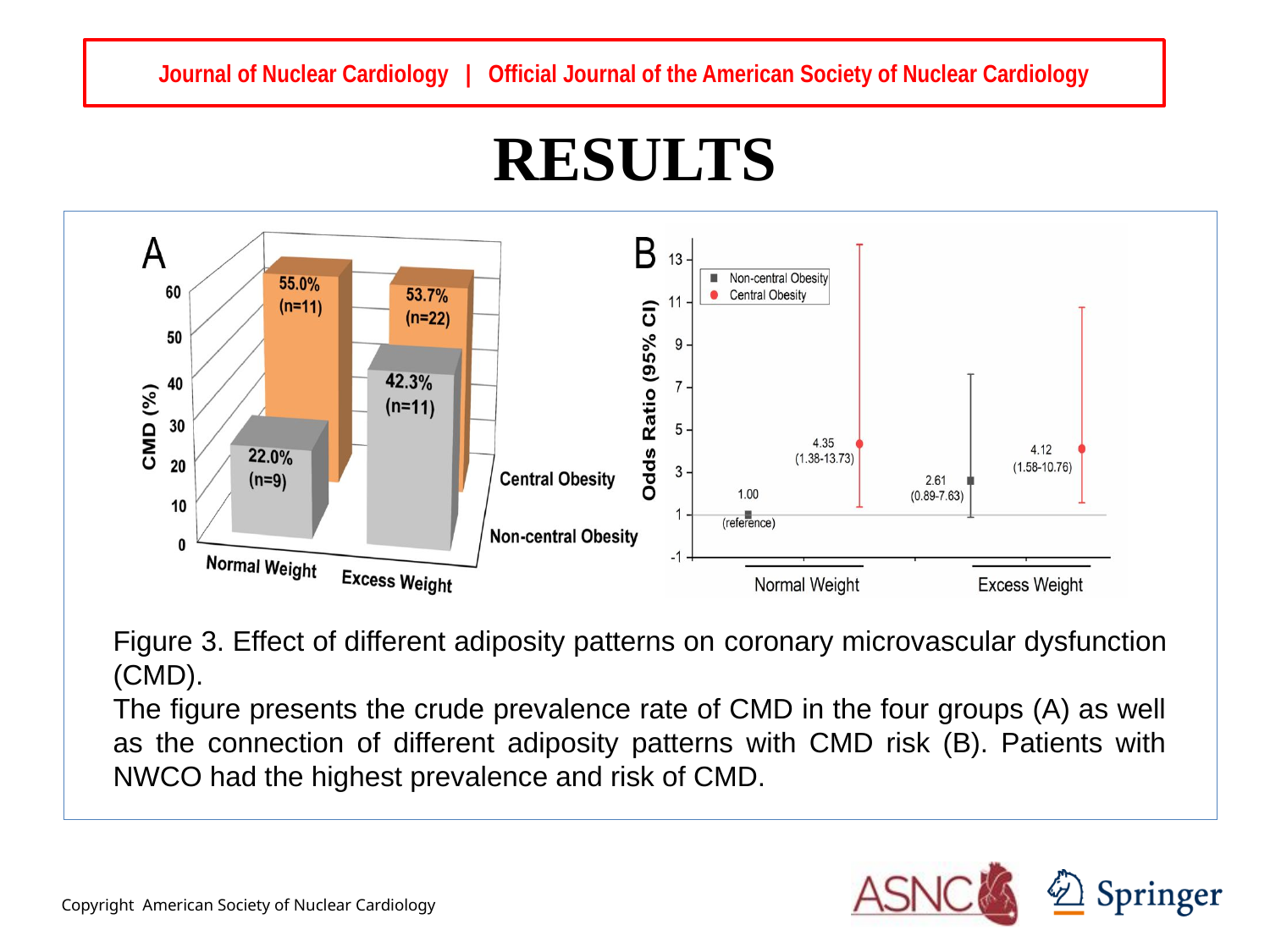

Journal of Nuclear Cardiology | Official Journal of the American Society of Nuclear Cardiology
# RESULTS
Figure 3. Effect of different adiposity patterns on coronary microvascular dysfunction (CMD).
The figure presents the crude prevalence rate of CMD in the four groups (A) as well as the connection of different adiposity patterns with CMD risk (B). Patients with NWCO had the highest prevalence and risk of CMD.
Copyright American Society of Nuclear Cardiology

## Slide 6
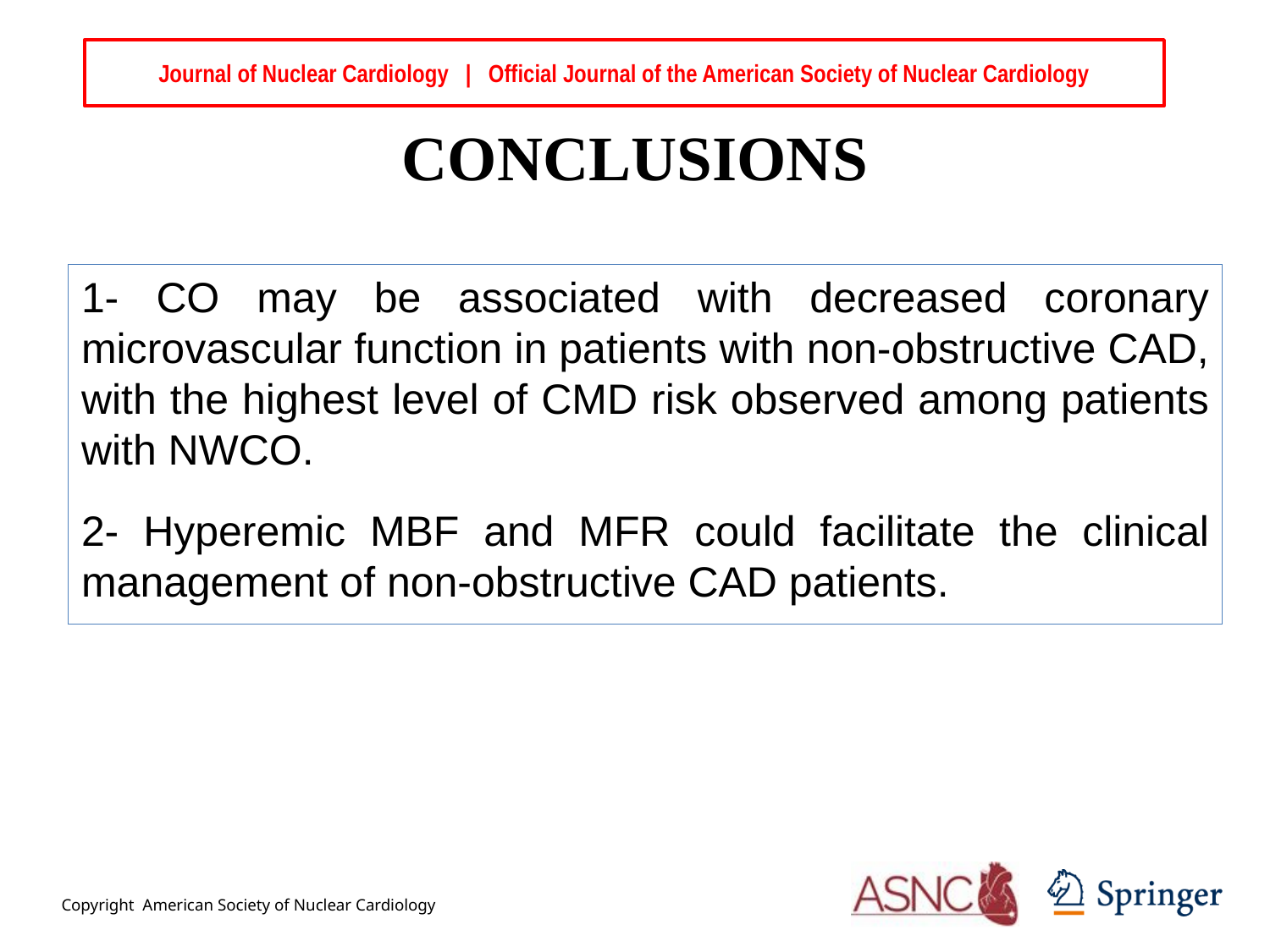

Journal of Nuclear Cardiology | Official Journal of the American Society of Nuclear Cardiology
# CONCLUSIONS
1- CO may be associated with decreased coronary microvascular function in patients with non-obstructive CAD, with the highest level of CMD risk observed among patients with NWCO.
2- Hyperemic MBF and MFR could facilitate the clinical management of non-obstructive CAD patients.
Copyright American Society of Nuclear Cardiology
